# Quality education from the perspective of landsenses ecology: research on performance evaluation of L2 teaching materials based on Importance-Performance Analysis

**DOI:** 10.3389/fpsyg.2026.1901977

**Published:** 2026-10-01

**Authors:** Lan Zhang

**Affiliations:** Department of Foreign Language, Mudanjiang Medical University, Mudanjiang, China

**Keywords:** Importance-Performance Analysis, landsense indicators, learning efficiency, linguistic landsenses, teaching material performance

## Abstract

At present, the evaluation of teaching materials mostly remains at the one-way comparison of indicators, lacking an effective mechanism to transform user feedback into improvement paths. Based on a questionnaire survey of L2 learners, this study aims to construct a dual mechanism: firstly, Importance-Performance Analysis is applied to identify the dimensions that need to be prioritized for improvement in the target teaching materials, and then mediation analysis is conducted to reveal the psychological pathways through which teaching materials influence learning outcomes. The results show that the perceived performance of landsense indicators is significantly lower than their importance, revealing a systematic performance gap that traditional unidimensional evaluation fails to capture. Furthermore, teaching material performance is positively correlated with foreign language enjoyment, and foreign language enjoyment is positively correlated with learning engagement. Learning engagement is further positively correlated with learning efficiency. This chain relationship provides statistical support for the process by which teaching material performance affects learning efficiency, revealing the vital role of the emotion-behavior chain mediation path in the transmission of teaching material effects.

## Introduction

Quality education is a core goal of the United Nations 2030 Agenda for Sustainable Development, and effective teaching materials are the foundation for achieving this goal, directly influencing learners' engagement and sense of efficacy. Second language (L2) teaching materials are the main medium for learners to come into contact with the target language, providing teachers and learners with progressive language input of course content and teaching guidance. Although research on L2 teaching materials has been extensive, encompassing both theoretical developments (Breen and Candlin, 1987; Hutchinson and Waters, 1987) and scale constructions (Mukundan and Ahour, 2010; Shak et al., 2022; Zhao, 1998; Chen et al., 2025), the dominant paradigm remains a static, one-way comparison of indicators. This approach is characterized by a singular methodology and a lack of systematic investigation of users' subjective experiences, making it difficult to effectively integrate the dynamic perceptions and genuine needs of end-users.

This disconnect has given rise to a pressing practical issue, namely the lack of an effective mechanism to transform quantified user feedback into practical improvement strategies. Traditional matching assessment (Hutchinson and Waters, 1987) and even the development of modern multi-dimensional scales (Litz, 2005) have largely focused on whether teaching materials conform to linguistic theories or syllabus requirements. However, they seldom investigate whether the priorities set by teaching material compilers and assessors align with what users deem important or satisfactory. Consequently, there is currently a lack of dynamic evaluation models and effective analytical methods that can facilitate interaction and communication among the needs, preferences, or visions of the multiple stakeholders involved in teaching materials (i.e., compilers, assessors, and users). This leaves teaching material revision without evidence-based, scientifically informed guidance derived from user data.

To address this gap, the present study introduces a brand-new dynamic evaluation framework that integrates user-centered quantitative diagnosis with impact pathway analysis. Grounded in the landsense concept and a learner-based evaluation index system for L2 teaching materials, the study employs Importance-Performance Analysis (IPA) to explore the needs, visions, and interactions among the multiple stakeholders involved in teaching material development. The aim is to generate insights for constructing teaching attribute profiles that support the development of teaching material users, as well as indicators for textbook quality assessment. We argue that moving beyond static scale-based evaluation (Liang, 2023) requires a two-step empirical approach: first, diagnose the specific strengths and weaknesses of teaching materials from the users' perspective; and second, reveal the psychological mechanisms through which teaching material quality influences learning outcomes. This approach provides the key operational methods and analytical tools for the paradigm shift in teaching material evaluation from entity research (focusing on disciplinary knowledge) to subjectivity research (focusing on the practical characteristics of teaching and learning) (Wu and Wang, 2021).

The significance of this study lies in its contribution to achieving a cross-level integration from micro-level diagnosis to macro-level impact. At the theoretical level, it moves beyond traditional static evaluation by constructing a dynamic model that integrates real-time diagnosis with impact mechanism analysis, offering a new framework for understanding how teaching materials truly work. At the practical level, it transforms large-scale user data into actionable priority improvement lists, providing direct tools for precise teaching material revision and scientific evaluation. By empirically revealing the core emotion-behavior learning pathway, it can provide an evidence base and theoretical foundation for empowering learner-centered educational decision-making and advancing the sustainable development of quality education.

## Theoretical basis and research hypothesis

### Landsenses ecology and linguistic landsenses

Landsenses ecology is a multidisciplinary theoretical system and research method proposed by Chinese ecologist Jingzhu Zhao, who defined it as a scientific discipline that studies land-use planning, construction, and management toward sustainable development, based on ecological principles and the analysis framework of natural elements, physical senses, psychological perceptions, socio-economic perspectives, process-risk, and associated aspects (Zhao et al., 2016). Its interdisciplinary integration and research methods based on people's multi-sensory and multi-dimensional perception have enabled it to be widely applied in fields such as urban construction (e.g., Zhang et al., 2023), community planning (e.g., Han et al., 2021), cultural communication (e.g., Zhao and Xiao, 2020), and educational guidance (e.g., Tarsitano et al., 2021). This theory holds that in social practice, people usually give or integrate one or more of their own visions into a carrier in an appropriate way, so that people (including themselves) can understand this or these visions in the carrier and its related manifestations, and these visions can guide or standardize people's words and deeds, so as to promote the realization of sustainable development (Zhao et al., 2020). The carrier of vision is called the landsense, and the process of conceiving and constructing a landsense is called landsense creation (Zhao et al., 2020). “These carriers can be hardware facilities, such as buildings, gardens, cities and blocks, as well as cultural resources such as poetry, novels, paintings, advertisements and songs” (Zhang et al., 2021a, p. 654).

The proposal of the concept of linguistic landsense further extends the idea of landsense from the physical space to the humanistic field of language use and language learning, giving rise to exploratory research on linguistic landsense in areas such as sustainable consumption behavior (Zhang et al., 2021a, 2025, 2026), ecological harmonious discourse analysis (Zhang et al., 2021b), linguistic ecology (Zhang et al., 2021c) and the complex language-behavior-environment system (Zhang et al., 2022; Zhang, 2024). Linguistic landsense is defined as “a meaning carrier of people's vision, which can integrate the designer's subjective vision into the appropriate linguistic carrier and play a role in cultural communication and educational guidance in real life” (Zhang et al., 2021b, p. 11). As a teaching tool that can make science education reform really enter the classroom (Wu and Wang, 2021), teaching materials, through their content organization, visual presentation, and structural design, constitute a significant source of users' multi-sensory experiences. They also serve as essential carriers through which multiple stakeholders (e.g., curriculum standard developers and teaching material designers) embed their pedagogical philosophies and curriculum visions. This positioning renders teaching materials a form of linguistic landsense, from which the users (teachers or students) of the teaching materials can understand the curriculum vision and teaching concepts bestowed by the designers, thereby promoting the users to achieve the prescribed curriculum goals and the sustainable development of science education reform. This aligns closely with the bidirection principle of vision manifestation emphasized within the landsense framework. This principle indicates that designers can integrate their own vision into a certain landsense, while contactors can grasp or understand the vision within the landsense (Zhao et al., 2020). In other words, the transmission of vision is not a one-way projection but is achieved through a two-way interaction where the designer endows it and the contactors understand it. In this process, the landsense serves as a key medium for the transmission of vision. In the context of teaching materials, this principle can be manifested as follows: the formulators of curriculum standards and the compilers of teaching materials constitute the vision endowing end, embedding curriculum concepts and teaching ideas into the content arrangement, visual presentation and structural design of teaching materials; teachers and learners constitute the vision receiving end, perceiving, understanding and internalizing the educational concepts and knowledge spectrum therein during the usage process. It is evident that teaching materials are neither pure containers of knowledge nor aimless formal designs, but rather landsense carriers that carry and convey the vision of the curriculum. The complete realization of their educational functions depends on the co-construction of meaning achieved through the interaction between the two ends of endowing and comprehension. Therefore, the research on the evaluation of teaching material landsenses from the perspective of landsenses ecology is not a replacement for the existing teaching material evaluation framework, but rather a systematic expansion of its assessment dimensions. The landsense evaluation can expand the evaluation of teaching materials from a one-way review of the text to a dynamic assessment of the vision transmission and meaning interaction among multiple subjects (e.g., curriculum standard formulators, teaching material compilers, teachers, and learners). This enables the evaluation of teaching materials to no longer be confined to the quality of the content, but to monitor whether the curriculum vision carried by the teaching materials has reached a consensus on meaning through the interaction of multiple subjects.

In addition, the framework and scale for textbook evaluation carry the assessors' and scale developers' cognition for the application value of the design elements of the textbook and their evaluation of the teaching function of the textbook. Thus, it also constitutes a linguistic landsense, and becomes a carrier and bridge for the demand response and vision resonance between the agents who tailor, compile or evaluate the teaching materials and the recepients who procure or use the teaching materials. However, to date, no research has systematically integrated the landsense concept with teaching material evaluation, either theoretically or empirically. In response to this situation, this study constructed a user-based landsense evaluation index system for L2 teaching materials (see Table A1 in Appendix), based on the curriculum design elements of the formulators, the educational concepts of the compilers, and the evaluation indicators of the assessors. IPA was then employed to diagnose the expectation-experience gaps in the teaching materials. Furthermore, a mediation model was used to examine the mechanism through which the performance of these landsense indicators influences learning efficiency. This study aims to provide a dynamic analytical framework that integrates user perception with empirical testing for teaching material evaluation, thereby filling a research gap in this field. Ultimately, it seeks to advance the research on teaching material evaluation from a superficial analysis of the content attributes toward an in-depth assessment of their subject attributes.

### Importance-Performance Analysis (IPA)

Importance-Performance Analysis (IPA), also known as importance-satisfaction analysis, is a comprehensive evaluation method proposed by Martilla and James (1977) for evaluating service quality and customer perception of service-oriented enterprises as well as the performance of various attributes. IPA mainly collects the data of the respondents' evaluation of the indicators of the specified survey object from two aspects of importance and satisfaction through questionnaire survey, thus forming the IPA matrix. In this matrix, the expectation degree (importance) of respondents to each indicator of the survey object is taken as the horizontal axis, and the satisfaction degree (performance) of respondents to each indicator is taken as the vertical axis, and the total average value of importance and performance is taken as the separation point of *X*–*Y* axis to construct a two-dimensional four-quadrant graph. The meanings of each quadrant are as follows: the quadrant I is the area of high importance and satisfaction, which is the dominant area, and the index factors falling into this area should be regarded as the key development objects; the quadrant II, with low importance and high satisfaction, is the retentive area, and a little attention can achieve better creation results; the quadrant III is a low area of importance and satisfaction, which is a slow improvement area, and the index factors falling into this area generally do not need to be considered; the quadrant IV, which has high importance but low satisfaction, is the key improvement area, and the index factors falling into this area can be used as breakthroughs to enhance the subjects' experience and should therefore be prioritized for improvement. Since proposed, IPA has attracted much attention from scholars and has been widely used in the fields of pedagogy (e.g., Zhang and Luo, 2022) and urban planning (e.g., Xu et al., 2017; Jiang et al., 2016), etc., but no scholars have applied it to the evaluation of teaching materials. In traditional paradigm of teaching material evaluation, scholars have predominantly employed a single-focus satisfaction judgment, drawing conclusions about how the textbook performs based on students' satisfaction ratings across various dimensions. While this approach captures students' intuitive responses, it fails to address a more critical question whether students' perceived performance aligns with their internal expectations. This is precisely where IPA innovates. By introducing importance as a reference coordinate, IPA expands the single-focus satisfaction evaluation into a dual-coordinate comparative analysis of expectation vs. experience.

Therefore, combined with research objectives and research questions, this paper uses IPA method (taking student satisfaction as teaching material performance) to evaluate the performance of textbook landsense indicators based on learner evaluation form. From the perspective of learners' perception, IPA method can be used to accurately and effectively identify the roles of landsense indicators of different dimensions in the high-quality development of teaching materials through the comparison of the two dimensions of learners' importance perception and satisfaction evaluation, which is highly innovative, practical and feasible.

### Learning efficiency

Learning efficiency refers to the effects and gains of learning activities engaged by learners within a defined timeframe, including learners' learning outcomes, learning performance and achievement, such as academic knowledge, scientific attitude, problem-solving ability, learning quality, experimental skills, values, etc. (Li et al., 2026). As an important indicator for measuring the degree of achievement of learning goals, learning efficiency not only reflects the effectiveness of an individual's learning process, but also reveals the practical utility of instructional design, learning materials and educational technology (Müller and Wulf, 2020; Ulrich et al., 2021). Some factors affecting learning efficiency have also been extensively explored. For example, Liu et al. (2021) revealed that the main factors affecting college students' learning efficiency include personal factors, family factors, school level factors, and so on. Among them, personal factors mainly include students' learning motivation and learning interest. Learning motivation can become a kind of learning power and have a positive impact on students' learning attitude and learning diligence. Kuh (2009) believed that the amount of time and energy students spent in the learning process had a lot to do with their academic performance.

In addition to learning performance such as grades and skill exercises, learning efficiency can also be reflected through the perception of students (Liaw and Huang, 2016). Research shows that perceived satisfaction and perceived usefulness are the key variables influencing learners' technology acceptance behavior (Liaw and Huang, 2007; Sun et al., 2008). The effectiveness of interactive learning environments is critical to helping learners communicate and share knowledge, and also has an important impact on enhancing positive attitudes such as perceived satisfaction and the usefulness of learning technologies (Liaw and Huang, 2007). From the perspective of landsenses ecology, teaching materials, as structured and systematic carriers of disciplinary knowledge, undertake the function of imparting knowledge and skills. Their teaching attributes inherently contain the potential to build an interactive learning environment. The integrity principle of psychological perceptions emphasized in landsenses ecology, reveals the internal mechanism by which an individual's multi-dimensional perception of the environment is integrated to form an overall psychological judgment. Similarly, learners' perceptual experiences regarding various landsense indicators of teaching materials (e.g., content appropriateness, structural clarity, and visual comfort) do not exist in isolation; rather, they intertwine during cognitive processing and are ultimately integrated into an overall evaluation of teaching material performance. This holistic performance identified through IPA, reflecting learners' integrated satisfaction with their multi-sensory and multidimensional experiences of landsense indicators for teaching materials, may directly shape their overall perceptual judgment of learning efficiency. Therefore, the following hypothesis is proposed:

H_1_: The performance of landsense indicators of teaching materials has a significant positive predictive effect on learning efficiency.

### Learning engagement

Learning engagement (ENG) refers to the state in which learners are actively engaged through high concentration of attention (Chiu, 2022; Lambert et al., 2017), involving determination of learning goals, participation in learning activities and positive attitude. As a dynamic construct (Appleton et al., 2008), the diachronic changes, multidimensional influencing factors, and associated effects of learning engagement have become a focal point of scholarly attention. Sulis (2022) highlighted that learning engagement was a process of continuous development and change, which presented obvious differences in different time scales or analysis levels. Liu et al. (2021) also pointed out that learning engagement could predict learning outcomes. Wang et al. (2022) further proved that learning engagement would have a certain impact on students' academic performance and personal development. The academic community generally regards learning engagement as a comprehensive construct, including behavioral engagement (i.e., students' participation in learning activities), emotional engagement (i.e., students' positive emotional experience), agentic engagement (i.e., students' enthusiasm and initiative in teaching activities) and cognitive engagement (students' cognitive effort in learning activities) (Reeve and Tseng, 2011). Reeve (2013) pointed out that these four dimensions collectively reflect students' capacity to achieve academic progress through behavioral engagement, emotional regulation, constructive participation in the instructional process, and the use of learning strategies.

Some researches show that compared with teacher-generated content, learning tasks involving student-generated content can better stimulate students' interest and engagement in multi-level learning (Lambert et al., 2017; Lambert and Zhang, 2019). This discovery reveals the significance of the interactive experience between learners and the learning content. From the perspective of landsense ecology, people's perception of the environment follows the multiscale principle of spatiotemporal combination (Zhao et al., 2020). In other words, this perception not only occurs within an immediate instantaneous field but also manifests as multi-level dynamic integration across temporal extension and spatial transition. Applying this principle to the field of teaching material evaluation, teaching materials are not only the medium for presenting knowledge, but also a learning environment field composed of multi-dimensional perceptual elements. From the micro-level (e.g., layout design, tactile sensation of fonts) to the meso-level (e.g., unit structure, content progression), and further to the macro-level (e.g., sustained use across a semester), learners' interactive experiences with teaching materials across different spatial and temporal scales collectively shape their multidimensional perceptions of teaching material performance. Some landsense elements (e.g., the applicability of the materials, structural coherence, and aesthetic design) serve as concrete manifestations of this spatiotemporally integrated perception, jointly influencing learners' emotional arousal and behavioral engagement during the learning process. The key to understanding whether teaching materials can truly stimulate learning engagement lies in effectively identifying learners' importance ratings and performance evaluations of each dimension of landsense indicators and diagnosing the potential gaps between them.

IPA introduced in this study helps to compare learners' importance ratings with their actual performance perceptions across various dimensions, promoting the effective identification of dimensions that learners highly value but experience poorly. This expectation–experience discrepancy essentially reveals the dysfunction of teaching materials as a learning environment field. When teaching materials fail to meet learners' expectations on key dimensions, their affordance and motivational force as environmental stimuli diminish, potentially inhibiting learners' willingness to engage and their level of effort. This constitutes the core mechanism for understanding how teaching material performance influences learning engagement. Based on this, the following hypotheses are proposed:

H_2_: The performance of landsense indicators of teaching materials has a significant positive impact on learning engagement.

H_3_: Learning engagement has a significant positive impact on learning efficiency.

### Foreign language enjoyment

Foreign language enjoyment (FLE) refers to the positive emotions that learners feel when learning a foreign language (Dewaele and Macintyre, 2014), involving FLE-Private (positive emotions stimulated by factors such as academic progress), FLE-Teacher (positive emotions stimulated by factors such as teacher support and encouragement) and FLE-Atmosphere (positive emotions associated with participation in class or group activities) (Li et al., 2018), characterized by a sense of novelty and accomplishment (Csikszentmihalyi, 2008). Enjoyment is an activity-centered emotion that is actively activated and has been shown to have a positive impact on learners' academic performance (Pekrun et al., 2007; Piniel and Albert, 2018) and the construction of language learning resources and broadening one's horizon (Macintyre and Gregersen, 2012). In addition, FLE can also strengthen language input or output ability, promote mental resilience, and improve learning efficiency. FLE has dynamic characteristics (Dewaele et al., 2022; Elahi Shirvan and Taherian, 2018), which has potential relationship with various social and psychological variables involved in foreign language learning, such as learners' self-evaluation and enjoyment, classroom anxiety (De Smet et al., 2018) and affection (Pavelescu and Petrić, 2018).

Thus, learners' perceptions of teaching materials are not merely simple mappings of their physical attributes (e.g., content, structure, form, and function) but rather integrated psychological constructs formed on the basis of multi-sensory perception and processed through individual cognitive schemas. For instance, the visual reception of layout design and color combinations can directly influence learners' first impressions and reading experience; the tactile experience of paper texture affects comfort and sense of affinity; spatial cognition regarding the structural organization or information hierarchy of the materials influences the efficiency of information retrieval and cognitive load; and the auditory perception of accompanying audio materials can also impact the sense of fluency and achievement in the learning process. This process of perception formation—integrating multiple physical sensory inputs (e.g., visual, auditory, and tactile sensations) with diverse psychological cognitions (e.g., attitudes, emotions, and intentions)—is fundamentally aligned with the core proposition of landsense ecology, namely the interactivity principle of physical senses and psychological perceptions (Zhao et al., 2020).

This principle emphasizes that physical senses and psychological perceptions are not isolated from one another; rather, they interact to jointly shape people' environmental experiences. People receive environmental information through multiple sensory channels, and this information is transformed into emotional experiences and behavioral intentions through cognitive processing. Extending this principle to the field of teaching material evaluation, teaching materials are not merely a medium for transmitting knowledge, but also a perceptual environment with which learners interact. Their design philosophy (e.g., visual layout), content presentation (e.g., language difficulty, example appropriateness), and cultural relevance (e.g., familiarity and identification with cultural elements) can all be regarded as environmental information acting upon learners' multi-sensory channels. Through learners' perception and cognitive processing, this information may evoke positive emotional responses, namely the experience of foreign language enjoyment. This enjoyment, triggered by the teaching materials, forms an emotional bond between learners and the materials, which may in turn strengthen the intensity of learning engagement and enhance the perceived level of learning efficiency. Therefore, the following hypotheses are proposed:

H_4_: The performance of landsense indicators of teaching materials has a significant positive impact on FLE.

H_5_: FLE has a significant positive impact on learning engagement.

H_6_: FLE has a significant positive impact on learning efficiency.

### Construction of landsense evaluation index system of teaching materials

The construction of the teaching material evaluation system in this study is based on the theory of landsenses ecology. This theory emphasizes that individuals receive environmental information through multiple senses and integrate it into an overall experience through psychological cognitive processing. When this theory is applied to the field of teaching material evaluation, teaching materials can be regarded as the perceptual field of learners, and their attributes (e.g., content, structure, and form) jointly constitute the environmental information that acts on learners' multi-sensory experiences. Based on this, the development and validation of landsense index system in this study follow a strategy that combines top-down and bottom-up approaches. This process not only deduces the perception dimension of teaching materials based on the theoretical framework of landsenses ecology, but also summarizes and supplements the indicators based on learners' actual experiences. At the top-down level, the research results of scholars (e.g., Zhao, 1998; Liang, 2023) on the evaluation of L2 teaching materials were systematically sorted out, and the dimension structure of teaching material evaluation scales at home and abroad was referred to. The core assessment dimensions of teaching material quality were initially extracted. At the bottom-up level, we have supplemented and adjusted the initial dimensions in combination with the demand levels and usage experience feedback of L2 learners. To ensure the content validity of the index system, two experts in the field of L2 teaching were invited to conduct two rounds of expert reviews on the initial index system. Based on expert feedback, the expressions of some indexes have been revised.

Ultimately, the evaluation index system was determined to include 10 element-level evaluation indicators such as student applicability, framework structure, theming content and language content (Table 1), involving a total of 39 index-level evaluation indicators (see Table A1 in Appendix). The design logic of this index system is rooted in the core tenets of the landsense concept, which is to comprehensively capture the experiential feedback elicited by teaching materials as a perceptual field for learners, starting from their multi-sensory and multi-dimensional experiences. Among them, multi-sensory experiences primarily include visual (e.g., layout, illustrations) and tactile (e.g., paper texture, binding) dimensions, etc., while multi-dimensional experiences encompass teaching material attributes such as content, structure, and format. Each indicator factor was scored on a Likert five-point scale (1 = very not important or very not dissatisfied and 5 = very important or very satisfied).

**Table 1:** The importance-performance means in the index level.

Element-level	Index-level	Importance (I)			Performance (P)			*I-P*	*T*	Sig. (two-tailed)	Adj. sig.
		*M* ±SD	95% CI	No.	*M* ±SD	95% CI	No.				
Student applicability	1	4.66 ± 0.65	[4.59, 4.73]	1	4.39 ± 0.78	[4.30, 4.47]	3	0.27	5.775	< 0.001	0.0167
	2	4.61 ± 0.63	[4.55, 4.67]	2	4.44 ± 0.83	[4.35, 4.53]	1	0.17	3.594	< 0.001	0.05
	3	4.55 ± 0.69	[4.48, 4.63]	4	4.38 ± 0.79	[4.29, 4.46]	4	0.18	3.788	< 0.001	0.025
	4	4.52 ± 0.73	[4.44, 4.60]	6	4.23 ± 0.93	[4.12, 4.33]	32	0.29	5.835	< 0.001	0.0125
Framework structure	5	4.47 ± 0.76	[4.39, 4.55]	19	4.37 ± 0.84	[4.28, 4.46]	5	0.10	2.059	0.040	0.025
	6	4.53 ± 0.70	[4.45, 4.60]	5	4.36 ± 0.80	[4.27, 4.44]	6	0.17	3.434	< 0.001	0.0125
	7	4.48 ± 0.75	[4.40, 4.57]	16	4.33 ± 0.86	[4.24, 4.42]	8	0.15	3.118	0.002	0.0167
Theming content	8	4.49 ± 0.75	[4.41,4.57]	15	4.21 ± 0.91	[4.11, 4.31]	35	0.27	5.524	< 0.001	0.0125
	9	4.46 ± 0.79	[4.37, 4.54]	25	4.27 ± 0.89	[4.17, 4.36]	26	0.19	4.053	< 0.001	0.0167
	10	4.45 ± 0.79	[4.36, 4.53]	28	4.28 ± 0.89	[4.18, 4.38]	18	0.17	3.350	< 0.001	0.025
Language content	11	4.46 ± 0.76	[4.38, 4.54]	23	4.29 ± 0.91	[4.20, 4.39]	15	0.17	3.149	0.002	0.05
	12	4.58 ± 0.65	[4.51, 4.65]	3	4.43 ± 0.78	[4.34, 4.51]	2	0.15	3.527	< 0.001	0.025
	13	4.50 ± 0.72	[4.42, 4.58]	12	4.32 ± 0.90	[4.22, 4.41]	9	0.18	3.653	< 0.001	0.0167
	14	4.52 ± 0.76	[4.44, 4.60]	7	4.29 ± 0.87	[4.19, 4.38]	17	0.23	5.000	< 0.001	0.0083
	15	4.52 ± 0.75	[4.44, 4.60]	8	4.30 ± 0.84	[4.21, 4.39]	14	0.22	4.527	< 0.001	0.01
	16	4.50 ± 0.80	[4.41, 4.58]	14	4.31 ± 0.86	[4.21, 4.40]	11	0.19	3.738	< 0.001	0.0125
Western culture	17	4.41 ± 0.80	[4.32, 4.49]	37	4.27 ± 0.86	[4.17, 4.36]	25	0.14	2.690	0.008	0.0167
	18	4.36 ± 0.85	[4.27, 4.45]	38	4.25 ± 0.87	[4.16, 4.35]	27	0.11	2.089	0.037	0.025
	19	4.33 ± 0.85	[4.24, 4.43]	39	4.23 ± 0.89	[4.13, 4.33]	30	0.10	1.972	0.049	0.05
	20	4.45 ± 0.77	[4.37, 4.54]	27	4.28 ± 0.86	[4.19, 4.38]	20	0.17	3.519	< 0.001	0.01
	21	4.47 ± 0.73	[4.39, 4.55]	20	4.31 ± 0.85	[4.22, 4.40]	10	0.16	3.201	0.002	0.0125
Activity design	22	4.48 ± 0.85	[4.39, 4.57]	17	4.22 ± 0.94	[4.12, 4.33]	33	0.25	4.850	< 0.001	0.01
	23	4.43 ± 0.83	[4.34, 4.52]	31	4.21 ± 0.91	[4.11, 4.31]	36	0.23	4.359	< 0.001	0.0125
	24	4.43 ± 0.79	[4.35, 4.52]	30	4.23 ± 0.93	[4.13, 4.33]	31	0.21	3.773	< 0.001	0.0167
	25	4.51 ± 0.79	[4.43, 4.60]	10	4.25 ± 0.94	[4.14, 4.35]	28	0.27	5.016	< 0.001	0.0083
	26	4.50 ± 0.73	[4.42, 4.58]	11	4.33 ± 0.86	[4.24, 4.42]	7	0.17	3.533	< 0.001	0.05
	27	4.46 ± 0.79	[4.37, 4.54]	24	4.28 ± 0.90	[4.18, 4.38]	21	0.18	3.610	< 0.001	0.025
Strategy training	28	4.51 ± 0.76	[4.43, 4.60]	9	4.27 ± 0.91	[4.17, 4.37]	24	0.25	5.085	< 0.001	0.0167
	29	4.46 ± 0.80	[4.38, 4.55]	21	4.28 ± 0.87	[4.19, 4.38]	19	0.18	3.512	< 0.001	0.05
	30	4.47 ± 0.77	[4.39, 4.56]	18	4.29 ± 0.89	[4.19, 4.39]	16	0.18	3.680	< 0.001	0.025
Localized content	31	4.46 ± 0.79	[4.38, 4.55]	22	4.27 ± 0.91	[4.17, 4.37]	22	0.19	3.627	< 0.001	0.025
	32	4.46 ± 0.78	[4.37, 4.54]	26	4.30 ± 0.92	[4.20, 4.40]	12	0.15	2.920	0.004	0.05
	33	4.50 ± 0.73	[4.42, 4.58]	13	4.22 ± 0.91	[4.12, 4.32]	34	0.28	5.414	< 0.001	0.0167
Formal device	34	4.43 ± 0.77	[4.35, 4.51]	32	4.23 ± 0.91	[4.13, 4.33]	29	0.20	3.879	< 0.001	0.0167
	35	4.42 ± 0.80	[4.33, 4.50]	35	4.30 ± 0.91	[4.20, 4.40]	13	0.12	2.245	0.025	0.05
	36	4.42 ± 0.84	[4.33, 4.51]	34	4.27 ± 0.93	[4.17, 4.37]	23	0.15	2.817	0.005	0.025
Supporting resources	37	4.41 ± 0.83	[4.32, 4.50]	36	4.12 ± 1.06	[4.00, 4.23]	39	0.29	4.976	< 0.001	0.0167
	38	4.42 ± 0.81	[4.33, 4.51]	33	4.14 ± 1.05	[4.02, 4.25]	38	0.28	4.749	< 0.001	0.025
	39	4.44 ± 0.87	[4.34, 4.53]	29	4.16 ± 1.06	[4.04, 4.27]	37	0.28	4.503	< 0.001	0.05

The Index-level column presents concise descriptors. The complete original survey items (in English) for all 39 indicators are listed in Table A1 (Appendix).

In the questionnaire, five items to measure FLE were selected from the study of Li et al. (2018), eight items to measure learning engagement were adapted from the study of Jang et al. (2016), and five items to measure learning efficiency were selected from the study of Lin et al. (2023) (see Table A2 in Appendix). The Chinese version of learning engagement scale, learning efficiency scale and FLE scale adopted the translation-back translation method to translate the English scale into Chinese. After expert review and the pre-test of 20 students, the expressions of some items were slightly adjusted to ensure cultural adaptability. Eventually, the Likert five-point scoring system was used for all the three scales (1 = strongly disagree, 5 = strongly agree).

### Methods and analysis

The main body of this study is non-English major undergraduates in a Chinese university, and the research object is the New Generation of College English teaching materials published by Foreign Language Teaching and Research Press. A cluster sampling method was adopted. From September to October 2024, four intact classes were randomly selected as sampling units among second-year non-English major students at a university in China, and all students in these classes were included in the survey. With the consent of the course instructors, questionnaires were distributed and collected in class. The participant sample had sufficient experience with the target teaching material examined in this study, enabling them to provide valid evaluations across its various dimensions.

A total of 360 questionnaires were distributed and 345 questionnaires were recovered, including 18 invalid questionnaires and 327 valid questionnaires (107 males and 220 females), with an effective rate of 90.83%. Participants' ages ranged from 18 to 22 years (M = 18.84, SD = 0.71).

Subsequently, SPSS 29.0 was employed for descriptive statistics and IPA, while AMOS 24.0 was used to conduct confirmatory factor analysis to assess the discriminant and convergent validity of the scales. The mediation effects were tested using the PROCESS macro (Model 6).

## Results

### Validity and reliability tests

To ensure the validity of the subsequent analyses of this study (including the IPA analysis of the teaching material and the test of the mediating mechanism between variables), we first conducted confirmatory factor analysis and reliability assessment on the core scales.

#### Confirmatory factor analysis

To test the measurement structure of the four core latent variables in the theoretical model (i.e., teaching material performance, foreign language enjoyment, learning engagement, and learning efficiency), a confirmatory factor analysis was first conducted. Due to the large number of items in the teaching material performance scale (39 items), including all items as manifest variables would have resulted in an overly complex model with unstable parameter estimates. Therefore, based on the theoretical constructs, we averaged the item scores within each dimension to generate 10 item parcels (i.e., the 10 element-level indicators shown in Table 1), which served as observed indicators for the latent variable of teaching material performance. For foreign language enjoyment, learning engagement, and learning efficiency, all original items were directly used as observed indicators.

The results of the analysis showed that the hypothesized four-factor model fit the data well: χ^2^/df = 2.88, CFI = 0.929, TLI = 0.922, RMSEA = 0.076, and SRMR = 0.0403. As shown in Table 2, all observed variables had standardized factor loadings ranging from 0.721 to 0.91 on their respective latent constructs, all reaching statistical significance (*p* < 0.001). Furthermore, the composite reliability (CR) of all latent variables ranged from 0.911 to 0.965, which was much higher than the acceptable threshold of 0.7 (Hair et al., 2017), and the AVE value was between 0.673 and 0.734, all higher than the excellent criterion of 0.5 (Fornell and Larcker, 1981). These results indicate that each scale has extremely high internal consistency, and the measurement model has very ideal convergent validity.

**Table 2:** Composite reliability (CR) and Average Variance Extracted (AVE).

Latent variables	λ	CR	AVE
Teaching material quality	0.721, 0.828, 0.846, 0.854, 0.858, 0.867, 0.867, 0.891, 0.908, 0.91	0.965	0.734
Foreign language enjoyment	0.77, 0.777, 0.82, 0.838, 0.892	0.911	0.673
Learning engagement	0.805, 0.811, 0.813, 0.814, 0.819, 0.833, 0.841, 0.858	0.944	0.680
Learning efficiency	0.774, 0.84, 0.841, 0.87, 0.882	0.924	0.709

#### Reliability statistics

After establishing the validity of the measurement structure, we assessed the internal consistency reliability of each scale using Cronbach's alpha coefficients. As shown in Table 3, the reliability coefficients for all core variable scales and the 10 sub-dimensions of teaching material quality exceeded the commonly accepted threshold of 0.7, indicating a high degree of reliability in the measurements. Specifically, the reliability coefficients for the two overall scales—teaching material performance and importance perception—both surpassed 0.9, and the 10 subdimensions underlying them also demonstrated good reliability (α ranging from 0.743 to 0.894). The reliability coefficients for the mediator and dependent variable scales (i.e., foreign language enjoyment, learning engagement, and learning efficiency) were also at a high level above 0.9. These results indicate that the scales employed in this study possess excellent internal consistency reliability, and the data obtained are highly reliable, stably reflecting the target constructs and satisfying the requirements for subsequent data analysis.

**Table 3:** Results of reliability analysis.

Measurement construct	Number of items	α	
		Importance perception α	Performance perception α
Teaching material quality (total scale)	39	0.970	0.978
Sub-dimension 1	4	0.836	0.857
Sub-dimension 2	3	0.743	0.813
Sub-dimension 3	3	0.793	0.830
Sub-dimension 4	6	0.849	0.882
Sub-dimension 5	5	0.865	0.885
Sub-dimension 6	6	0.894	0.893
Sub-dimension 7	3	0.779	0.835
Sub-dimension 8	3	0.811	0.856
Sub-dimension 9	3	0.807	0.839
Sub-dimension 10	3	0.815	0.861
Foreign language enjoyment	5	0.902	
Learning engagement	8	0.943	
Learning efficiency	5	0.923	

### Descriptive statistics and correlation analysis

Table 4 listed the mean and standard deviation of each variable and their Pearson correlation coefficients, indicating that there were significant positive correlations among teaching material performance, FLE, learning engagement and learning efficiency (*P* < 0.001), and the correlation coefficients ranged from 0.333 to 0.854, among which the correlation between learning engagement and learning efficiency was the strongest. To systematically evaluate the impact of this high correlation and other potential methodological issues on subsequent regression analysis, common method bias tests and multicollinearity diagnoses were conducted, respectively.

**Table 4:** Descriptive statistics and correlation analysis of variables.

Variables	*M*	SD	Teaching material performance	Foreign language enjoyment	Learning engagement	Learning efficiency
Teaching material performance	4.28	0.66	1			
Foreign language enjoyment	4.21	0.78	0.333^**^	1		
Learning engagement	4.19	0.77	0.387^**^	0.788^**^	1	
Learning efficiency	4.02	0.87	0.366^**^	0.825^**^	0.854^**^	1

^**^Correlation is significant at the 0.01 (2-tailed).

### Testing for common method bias

All data for the core variables in this study were collected through student self-reports, so there was a potential risk of common method bias. To address this, we employed Harman's single-factor test, the most commonly used statistical diagnostic method. This test was conducted on the variables that were jointly analyzed in the subsequent mediation model. With the help of SPSS 29.0, all items in the questionnaire measuring teaching material performance, FLE, learning engagement and efficiency were used for exploratory factor analysis, and Harman single factor test was performed on the collected data. The items measuring the importance perception were not involved in the subsequent path analysis and their data were solely used for IPA, so they were excluded from this test. Results are as follows. The explanatory volume of the first factor was 42.58%, which was lower than the threshold (50%), indicating that the common method bias was acceptable (Hair et al., 1998).

### Collinearity diagnostics

To test for multicollinearity, all predictor variables in the mediation model (i.e., teaching material performance, foreign language enjoyment, and learning engagement) were included in a linear regression equation with learning efficiency as the dependent variable, and the variance inflation factor (VIF) and tolerance values (Tol) for each variable were calculated.

As shown in Table 5, the VIF for all predictor variables ranged from 1.179 to 2.767, which were below the critical threshold of 5, and the corresponding Tol all exceeded the common criterion of 0.1. This indicates that, despite the high bivariate correlation observed between learning engagement and learning efficiency, no severe multicollinearity issues were present in the multiple regression framework. Consequently, this does not pose a substantial threat to the parameter estimation of the subsequent mediation effects.

**Table 5:** Overall multicollinearity test.

Dependent variable	Independent variables	Tol	VIF
Learning efficiency	Teaching material performance	0.848	1.179
	Foreign language enjoyment	0.378	2.646
	Learning engagement	0.361	2.767

### IPA results

#### Descriptive analysis of the importance-performance of the index level

This study conducted an IPA on the 39 landsense indicators of the surveyed teaching material, aiming to diagnose their strengths and weaknesses from the learners' perspective.

According to the means of importance shown in Table 1, all 39 evaluation indicators have been agreed by learners, and all means are above 4 points, indicating that learners believe that the content of these 39 indicators has a significant impact on their English learning activities during the use of the tested teaching material. For each indicator, the mean of performance is slightly lower than the mean of importance although the mean performance for each indicator is also above 4 points, indicating that the teaching material tested in these indicators of the construction of learning is in place, and learners' satisfaction is relatively high, but there is still a certain room for improvement.

To assess whether there were statistically significant differences between the importance and performance ratings for each dimension, we conducted paired-sample *t*-tests. According to the initial results of paired *t*-test analysis, the importance-performance (I-P) differences of 39 indicators were all positive and above the significance level, of which 10 indicators (*P* < 0.05) had significant differences, and the remaining 29 indicators (*P* < 0.001) had extremely significant differences.

To control for the potential inflation of Type I errors resulting from 39 paired comparisons, we applied the Holm–Bonferroni method to adjust the raw *p*-values and calculated the adjusted *p*-values. As shown in Table 1, the I-P differences for all 39 sub-dimensions remained statistically significant after correction, indicating that the performance shortfalls were statistically reliable. Consequently, these dimensions should be regarded as key areas requiring attention and improvement in subsequent teaching material revision and resource allocation.

The above findings reveal that, based solely on a performance scale, learners' evaluation of the teaching material appeared satisfactory (with mean performance scores for each indicator ranging from 4.12 to 4.44). However, the two-dimensional comparison of IPA uncovers a more critical fact: learners' expectations across various dimensions of the teaching material exceeded their actual experiences, indicating a systemic performance-expectation gap in the textbook as a whole. This finding profoundly exposes the fundamental limitations of traditional evaluation paradigms of teaching materials. Whether it is the matching assessment based on expert checklists (Hutchinson and Waters, 1987) or the absolute value judgment relying solely on satisfaction surveys, both fail to capture learners' authentic experiences. The former, while capable of addressing theoretical compliance (e.g., verifying the alignment between teaching materials and predetermined checklists), cannot answer the core practical question of whether the textbook meets learners' expectations. The latter also has blind spots: without importance as a reference, it is impossible to identify the potential expectation gaps hidden behind seemingly satisfactory ratings.

This highlights the value of introducing IPA in this study. By incorporating importance as an evaluative dimension, IPA transforms static satisfaction assessments into a dynamic analysis of expectation-experience gaps, thereby revealing authentic issues that traditional methods fail to capture. This finding aligns closely with the call by Wu and Wang (2021) for a paradigm shift from entity research to subjectivity research in teaching material evaluation—that is, moving beyond static examinations of knowledge itself toward a focus on learners' practical perceptions and experiential gaps. Future research could integrate qualitative methods (e.g., interviews) to gain deeper insights into the reasons behind learners' evaluations of specific indicators, thereby providing richer evidence for the improvement of teaching materials.

#### IPA quadrant analysis

The IPA quadrant diagram was used to further analyze 39 indicators. In the IPA quadrant diagram (Figure 1), the mean of overall importance of 39 indicators (4.4744) and the mean value of overall satisfaction (4.2788) were taken as vertical crossing points, and the importance mean of indicators was taken as horizontal axis and performance mean as vertical axis to establish a four-quadrant matrix. This approach of employing grand means as intercepts follows the standard IPA procedure (Martilla and James, 1977), as it reflects the overall evaluation level of the sample and enables a natural identification of dimensions that are high or low relative to the overall mean. According to the data in Table 1, the IPA quadrantal distribution of performances of 39 indicators in the tested teaching material can be obtained (see Figure 1).

**Figure 1:**
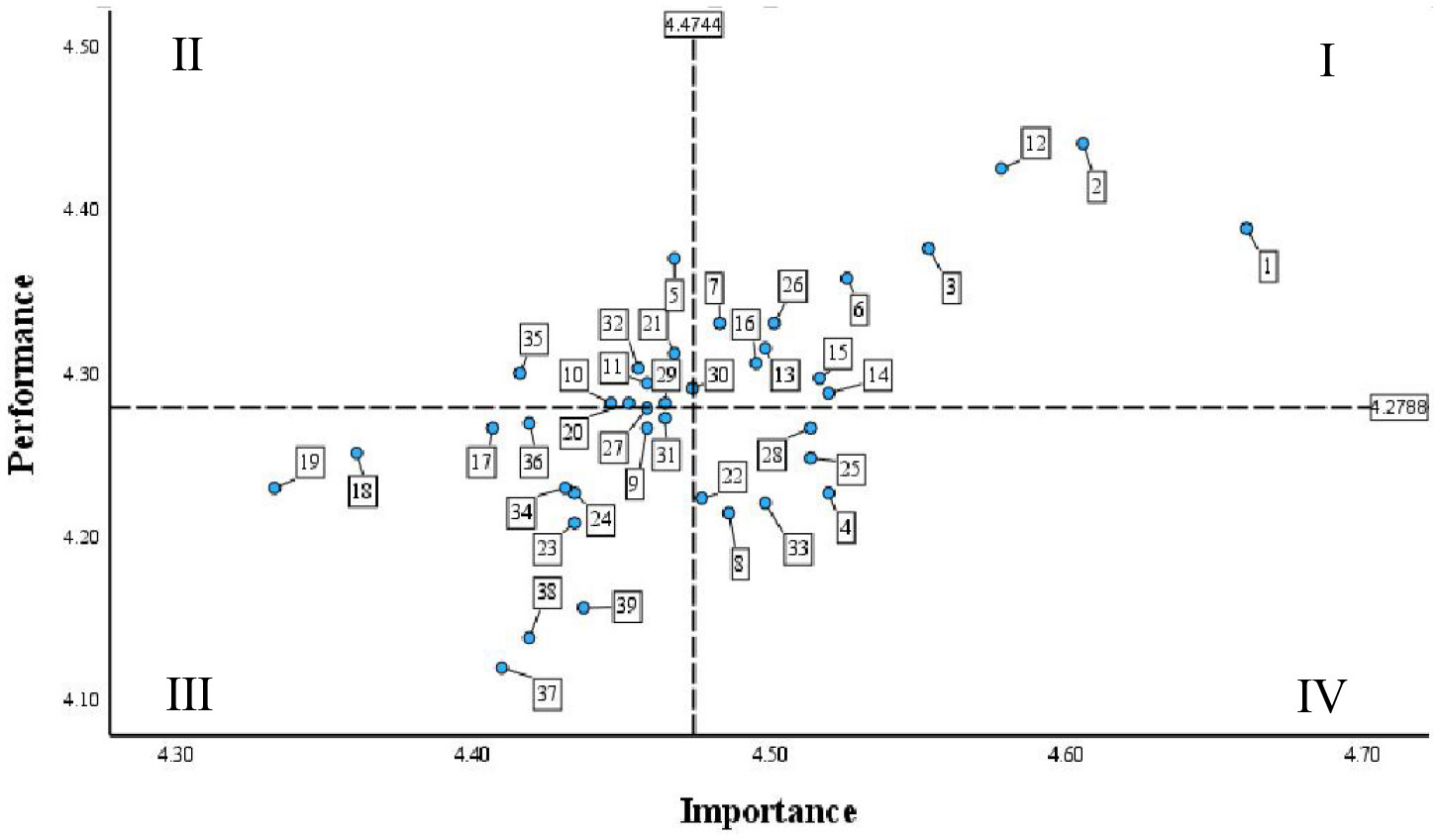
IPA quadrants of 39 indicators of teaching material performance. The origin of coordinates represents the grand means of importance (*M* = 4.4744) and performance (*M* = 4.2788). For the specific indicators corresponding to the numbered points in the figure, see Table A1 in Appendix.

As shown in the IPA quadrant diagram (Figure 1): the first quadrant contains 11 indicators, 5 of which belong to “language content” elements, indicating the repetition rate of new words, typical example sentences, the annotation and translation of new words and grammar in this series of teaching materials have been fully affirmed and paid attention to by learners; the second quadrant has five indicators, which are, respectively, “unit module design,” “language communication,” “content arrangement of Western culture,” “integration into Chinese culture,” and “illustration and content coordination,” indicating learners are satisfied with the actual perception of unit modules, communication and Chinese culture integration, but the index I-P difference is positive, which indicates that the content arrangement and appearance configuration can be further standardized and improved; there are 12 indicators in the third quadrant, 3 of which belong to Western culture and 3 of which belong to supporting resources; the fourth quadrant has six indicators, which are “the learning content arousing students' interest,” “diverse theme ortopics,” “activities or exercises integrating the use of English skills,” “provision of major language learning strategies,” “Chinese annotation,” etc., indicating that these indexes need to be improved urgently. The relationship between teaching material performance assessed by these indicators and FLE, learning engagement and learning efficiency should be verified by the following intermediary analysis results.

### Mediation analysis results

SPSS Process macros were used to examine the mediating effects of FLE and learning engagement on teaching material performance and learning efficiency through the non-parametric percentile Bootstrap method of mediating effect test. The regression analysis showed that the direct effect of teaching material performance on learning efficiency was significant before adding mediating variables. When FLE and learning engagement were included in the regression equation, teaching material performance had a significant positive effect on FLE and learning engagement, but no longer had a significant effect on learning efficiency. FLE had significant positive effects on learning engagement and learning efficiency, respectively. And learning engagement had a significant positive impact on learning efficiency, as shown in Table 6.

**Table 6:** Chain mediation analysis.

Regression equation		Overall fit index			Regression coefficient	
Outcome variable	Predictor Variable	*R*	*R* ^2^	*F*	β	*t*
FLE	TMP	0.33	0.11	20.42^***^	0.33	6.35^***^
ENG	TMP	0.80	0.64	191.41^***^	0.14	3.97^***^
	FLE				0.74	20.96^***^
EFF	TMP	0.89	0.79	309.43^***^	0.03	0.95
	FLE				0.39	9.51^***^
	ENG				0.53	12.67^***^

Each variable is normalized before being brought into the equation. ^*^p < 0.05, ^**^p < 0.01, and ^***^p < 0.001. FLE, Foreign language enjoyment; TMP, Teaching material performance; ENG, Learning engagement; EFF, Learning efficiency.

The intermediate effect test and confidence interval estimation were carried out for the three paths, respectively. The results showed that 95% confidence intervals of the three paths did not include 0, indicating that the indirect effects of the three paths had reached a significant level (as shown in Figure 2). The total standardized mediation effect was 0.338, accounting for 93.89% of the total effect. The indirect effects of the three intermediary paths accounted for 36.39%, 20.83%, and 36.67% of the total effects, respectively, as shown in Table 7.

**Figure 2:**
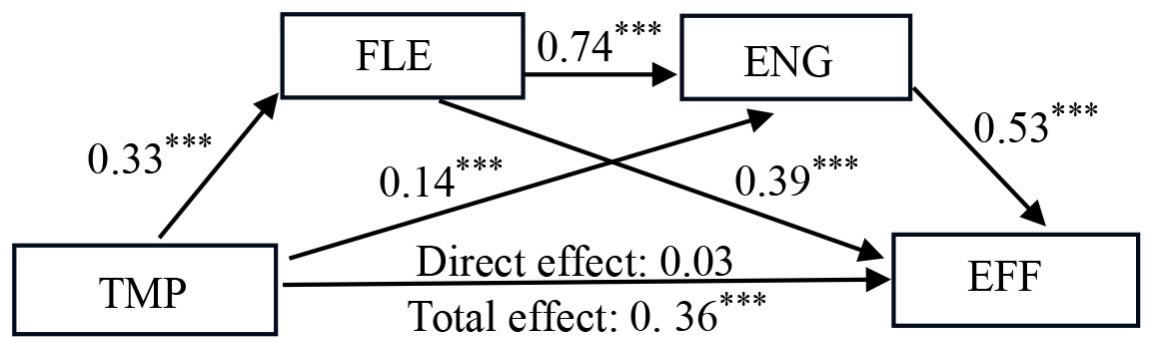
Model diagram of chain mediation effect. ^*^p < 0.05, ^**^p < 0.01, and ^***^p < 0.001. FLE, Foreign language enjoyment; TMP, Teaching material performance; ENG, Learning engagement; EFF, Learning efficiency.

**Table 7:** Mediating effects and confidence intervals.

Path	Effect size	Proportion of total effect	95% CI	
TMP → FLE → EFF	0.131	36.39%	0.085	0.190
TMP → ENG → EFF	0.075	20.83%	0.029	0.129
TMP → FLE → ENG → EFF	0.132	36.67%	0.085	0.189
Total indirect effect size	0.338	93.89%	0.236	0.461

CI, confidential intervals.

## Discussion

### The feedback adjustment mechanism of teaching material landsense among plural subjects

The United Nations Millennium Development Goals 4 (Quality Education) proposed to ensure inclusive and equitable quality education and promote lifelong learning opportunities for all. The construction and evaluation of teaching materials is a priority field of action to carry out quality education and a concrete way to promote sustainable development education. As an important medium through which learners are exposed to scientific knowledge, teaching materials need to provide students with engaging and academically challenging learning experiences and support their self-directed learning. Additionally, the chain mediating effect of “teaching material performance → foreign language enjoyment → learning engagement → learning efficiency” has been demonstrated in this paper, which indicates that teaching material performance can be an organic component of quality education. However, if a teaching material is only used as the carrier of knowledge and the objective object of students' learning, it has the entity characteristics independent of learners, and often lacks the subject relevance for learners, which is not conducive to the interaction between learners and the teaching material, difficult to help learners achieve their own academic development (Wu and Wang, 2021), and is not conducive to the realization of quality education goal. Therefore, how to enhance the analysis of teaching attributes and quality assessment of teaching materials has become an important issue that urgently needs to be addressed in the implementation of science courses and the use of teaching materials.

However, it is worth noting that the planning, design, management and evaluation of teaching materials is a comprehensive and complex process, and the implementation process of teaching materials in teaching practice does not only pursue a single goal or result, but also involves the comprehensive feedback and gradual adjustment of the design concept, evaluation ideas and learning needs among plural subjects (e.g., textbook designers, assessors, and users). This is in line with the overall meliorization framework (Zhao et al., 2021) of landsenses ecology, which uses the meliorization model and hyperfeedback mechanism to promote the sustainable development of complex systems. Therefore, based on landsenses ecology, this paper constructs a hyperfeedback adjustment mechanism for textbook designers, assessors and users (Figure 3) to analyze the vision resonance among plural subjects of teaching materials and its effect on the landsense creation of learners' learning engagement, enjoyment and efficiency.

**Figure 3:**
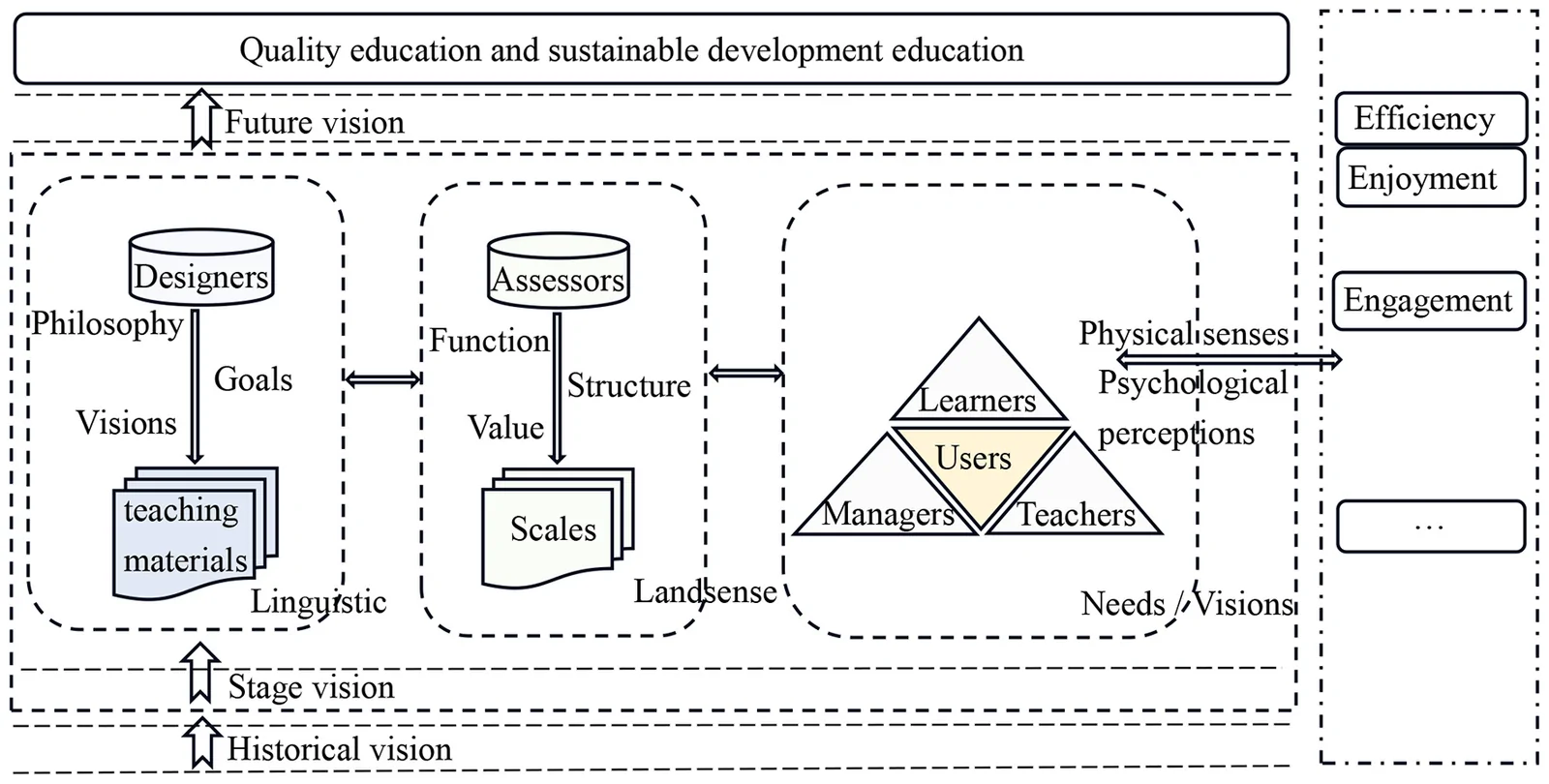
The overall meliorization framework and the hyperfeedback mechanism of plural subjects of teaching materials.

As shown in Figure 3, the teaching material designers integrate their understanding of the syllabus and visions of the teaching goal into the compilation and design of the textbook through appropriate forms of manifestation, with the aim of effectively stimulating the sensory system of learners, thus triggering a series of knowledge perception and psychological cognitive processes. The teaching material bearing the designers' design concept and visions constitutes a linguistic landsense, and the process of its landsense creation is closely related to the user's perceptions of the teaching material. In view of this, teaching material assessors usually start with the key links of teaching implementation, and conduct practice-oriented application value assessment on the design of teaching support elements in teaching materials, so as to comprehensively analyze and evaluate the teaching function of teaching materials. These reference elements, which are used to evaluate and judge the performance and level of teaching material in helping students learn, constitute the landsense indicators of teaching material evaluation, carrying the understanding and visions of assessors on the diversity and accessibility of learning opportunities for students in the teaching material. They usually focus on whether the teaching material can provide opportunities for students of different types or levels to actively participate in the learning by designing an attractive variety of content materials and practical activities. On the one hand, based on the evaluation of the cognitive value and application value or function of the teaching material, assessors give feedback on the application of the design concept and visions of the teaching material designers in real teaching scenarios; on the other hand, the problem scenarios in the textbook (e.g., the text content and practical activities), which are closely related to students, are organized and designed, so as to comprehensively and deeply evaluate the accessibility of the textbook from multiple perspectives (e.g., learners' cognition, behavior, and interaction), thus improving learners' learning engagement, enjoyment, efficiency, scientific literacy, and practical ability, etc. The main reason for the existence and development of teaching materials is that they provide convenient means for the systematic structure of teaching, but there are no perfect teaching materials (Zhang, 2001). Teaching material evaluation is really a process of alignment (matching needs with feasible solutions) (Qian, 1995). The meliorization framework and the hyperfeedback mechanism of plural subjects of teaching material emphasize that it is necessary to analyze each element of the process of knowledge perception and psychological cognition of teaching materials separately, and to take them as a whole to analyze their mutual influence and function. At the same time, it is necessary to integrate more adequate perception data from people. The operation process of the model is not a one-way optimization problem, but a complicated interweaving of constraints and objectives in each evaluation dimension (e.g., the possibility and quality level of teaching materials to support students and teachers to achieve curriculum literacy goals), and a process of improve step-by-step in each evaluation dimension or stage with the support of multi-source data. According to the different characteristics of the landsense indicators, there are different manifestations of the meliorization model, but its core idea is to emphasize the continuous feedback, regulation and improvement in the process of teaching material use and evaluation. Using the plural-subjects meliorization thinking to study the problem of teaching material evaluation will make the goal and process of textbook curriculum construction more clearly understood, and take timely countermeasures according to the actual application of teaching materials and the needs of users. The application of the meliorization thinking in the concept of landsenses ecology can make the landsense design and evaluation of teaching materials get rid of the once-and-for-all planning ideas, so as to better adapt to the sustainable development of teaching materials and meet the needs of learning subjects constantly updated.

### The application prospect of IPA and linguistic landsenses in teaching material evaluation

The traditional evaluation framework mainly relies on single-dimensional satisfaction judgments. By collecting learners' satisfaction scores for several dimensions of the teaching materials, it draws conclusions on how the teaching materials perform. Although this evaluation method can capture the learners' intuitive feelings, it cannot answer another more crucial question whether the performance perceived by the students matches their inner expectations. IPA adds the reference dimension of importance, expanding the single-dimensional satisfaction judgment to a two-dimensional comparison and analysis of expectations and experiences, thus transforming the evaluation of teaching materials from a static description of satisfaction levels to a dynamic diagnosis of whether expectations and experiences match. This discovery does not deny the value of the traditional framework, but rather points out that the satisfaction standards on which the traditional evaluation framework is based lack reference system calibration. Its judgment results may overestimate the experience quality of the teaching materials in actual use and lack the backtracking and correction mechanism starting from the learner's experience.

Furthermore, the IPA results of this study show that learners' evaluations of the importance and satisfaction of Western cultural elements are both at a relatively low level, indicating that there may be a mismatch between the designer's intention and the user's perception. This discovery proposes a further revision to the previous call that teaching material evaluation should shift from entity research to subjectivity research (Wu and Wang, 2021), that is, simply turning to users may not be sufficient. If the single-focus satisfaction survey is still used, even if it turns to users, only satisfaction data can be obtained, but the gap between importance and satisfaction cannot be identified. In other words, turning to users is only the first step. The key lies in what kind of analytical framework to introduce. The evaluation of teaching material landsense based on IPA is a systematic expansion of the existing assessment dimensions, transforming the evaluation of teaching materials from the static judgment of a single subject on the text to the dynamic analysis of the perceptual interaction of multiple subjects during the use of teaching materials. At the same time, it enables the revision of teaching materials to adopt a hierarchical action strategy: immediate revision items (indexes falling into the fourth quadrant of high importance but low performance), mid-term optimization items (indexes falling into the third quadrant of low importance and low performance), advantage maintenance items (indexes falling into the first quadrant of high importance and high performance), and moderate attention items (indexes falling into the second quadrant of low importance but high performance). In addition, the teaching material planning should follow the principle that multiple subjects jointly create landsense indexes. Before revision, the designers should conduct research on the current status of teaching materials and the users' demands, guiding the teaching material compilers and users to jointly participate in the planning and construction of teaching material indexes, so as to promote the positive transfer from in-class learning to extracurricular application, laying a foundation for the accumulation of students' humanistic qualities and the cultivation of their lifelong learning abilities.

## Conclusion and suggestion

### Summary of findings

This study, taking learners' perceptions as the point of departure, analyzes the landsense-oriented optimization model and hyperfeedback mechanism through which teaching material performance promotes learning engagement, enjoyment, and efficiency from behavioral, cognitive, emotional, and social dimensions. The main findings can be summarized into three aspects.

First, among the multiple dimensions of teaching material landsense, student applicability plays a dominant role in subjects' perceptual experience, followed by language content, while subjects pay relatively little attention to Western cultural elements. This finding reveals the hierarchical nature of textbook landsense—that is, learners' perceptions of teaching materials are not evenly distributed but are centered on the dimensions most relevant to their own learning practices. This provides specific directions for teachers to adjust input in order to facilitate learning engagement.

Second, the IPA analysis uncovered deep-seated issues that traditional satisfaction surveys fail to capture. By introducing importance as a reference coordinate, it extends the traditional unidimensional satisfaction evaluation into a dual-coordinate expectation-experience gap analysis, offering a more explanatory diagnostic tool for assessing teaching material quality.

Furthermore, the mediation analysis revealed that the influence of teaching material performance on learning efficiency can operate through both direct and indirect pathways. The direct effect validated the immediate impact of the teaching material as a learning environment field, while the indirect effect was realized through the sequential pathway of foreign language enjoyment and learning engagement. This finding corroborates the hierarchical transmission mechanism of “physical senses → psychological perceptions → behavioral intention” emphasized in the landsense concept. This constitutes the intrinsic mechanism through which the multiple landsense indicators of teaching materials affect learning outcomes.

### Theoretical contributions and practical implications

The theoretical contribution of this study lies in extending the landsense concept from the field of environmental perception to the domain of teaching material evaluation, thereby providing a new theoretical lens for understanding the subjective attributes of teaching materials. IPA further expands the evaluation of teaching materials to the analysis of the expectation-experience gap of landsense indicators. Its dual-coordinate positioning function can also answer the practical question of which gaps should be prioritized for improvement, achieving the transformation from problem diagnosis to action guidance. Additionally, a chain mediation model uncovers the psychological mechanism underlying the influence of teaching material performance on learning outcomes, offering empirical insights into how teaching materials truly function.

On a practical level, the value of this study is reflected in three dimensions. For teaching material editors, it provides an evidence-driven priority list, clarifying which aspects require priority improvement and which strengths should be maintained, thereby shifting teaching material revision from experience-based judgment to data-driven decision-making. For frontline teachers, the findings suggest that the key to enhancing learning efficiency lies not only in the teaching material content itself, but also in how instructional design and classroom interactions can activate the potential of teaching materials to stimulate positive emotions and promote active engagement. For educational policymakers, this study offers a dynamic analytical tool for teaching material evaluation, laying a methodological foundation for constructing an evaluation community involving multiple stakeholders (e.g., editors, assessors, and users).

### Limitations and suggestions

Several limitations of this study should be acknowledged. First, the sample was drawn from L2 learners at a specific university, whose educational background and cultural context have certain particularities; thus, the cross-contextual generalizability of the findings requires further examination. Second, this study employed cross-sectional data, which can only capture users' perceptions at a specific point in time and cannot reveal the dynamic evolutionary patterns of expectation-experience gap of landsense indicators during the process of teaching material use. Third, this study focused on a single teaching material case. Although this allows for an in-depth analysis of its landsense characteristics, the generalizability of the conclusions needs to be verified through more empirical studies involving different teaching materials and disciplines.

Based on the aforementioned limitations and the findings of this study, future research could expand the heterogeneity of the sample to include learner groups from different educational stages, cultural backgrounds, and language proficiency levels, thereby examining the cross-group stability of the landsense perception structure and exploring the influence mechanisms of cultural context on teaching material evaluation. In addition, longitudinal designs could be employed to track the dynamic changes in learners' perceptions of teaching materials across different learning stages, revealing the evolutionary patterns of teaching material landsense and its temporal relationship with learning outcomes. Furthermore, integrating qualitative methods (e.g., interviews and focus groups) could provide deeper insights into the reasons behind learners' evaluations of specific indicators, combining quantitative diagnosis with qualitative understanding. Meanwhile, variables (e.g., teacher factors and classroom atmosphere), which may influence learning engagement and enjoyment, should also be incorporated to provide a richer evidence base for teaching material improvement.

## Data Availability

The original contributions presented in the study are included in the article/supplementary material. Further inquiries can be directed to the corresponding author/s.
