# Supplementary material for: Quality education from the perspective of landsenses ecology: research on performance evaluation of L2 teaching materials based on Importance-Performance Analysis
Source: Front Psychol. 2026 Oct 1;17:1901977. doi: 10.3389/fpsyg.2026.1901977 (PMC13668830; doi:10.3389/fpsyg.2026.1901977)
Supplement: Supplementary file 1 [file Table_1.pdf]

Appendix

Table A1. The complete survey items corresponding to the 39 sub-dimensions of teaching material quality.

Index-level	Full Item Description
1	Meet the student's foreign language level, with moderate difficulty.
2	Suitable for students' age and cognitive level.
3	Meet students' learning goals and needs.
4	Arouse students' interest in learning content.
5	The modules in each unit or lesson are well designed and interlinked.
6	The teaching content (including language knowledge, language skills and culture, etc.) has reasonable layout, close connection and moderate increase in difficulty.
7	Set up word lists, grammar lists, and other indexed lists easy for students to search.
8	Theme or topic is rich and varied, which meets the needs of students' real communication.
9	Theme or topic arrangement conforms to the learning rule from easy to difficult, step by step.
10	The subject matter of the text covers a wide range of genres.
11	Language is true, natural and communicative.
12	Language is normative and accurate.
13	The number of new words in each unit is appropriate, with full recurrence rate.
14	Provide example sentences for new words and grammar, and context if necessary, to make the context clearer.
15	The annotation and translation of new words and grammar are concise, accurate and easy to understand.
16	The proportion of listening, speaking, reading, writing and translating skills training is reasonable.
17	Contain the cultural content of western society and people's life.
18	Contain the cultural content of the excellent western tradition.
19	Contain cultural content of contemporary western national conditions.
20	Contain western cultural factors related to language use and communication.

21	The arrangement of western cultural content is reasonable and moderate, with a natural and appropriate way of presentation.
22	Types and forms of activities or exercises are varied.
23	Activities or exercises can help students to master the content effectively.
24	Activities or exercises can promote meaningful interaction.
25	Provide activities or exercises that integrate the use of English skills.
26	Activities or exercises have clear goals.
27	Instructions for activities or exercises are clear, concise and operable.
28	Provide key language learning strategies (e.g., conjecture, association, self-assessment, finding information, etc.) to help students better grasp the learning content.
29	Provide positive communication strategies (e.g., explanations, corrections, repeats, clarifications, requests, etc.) to help students better complete communicative tasks.
30	Provide listening strategies and listening skills training (e.g. listening to key points, taking notes, skimming information, etc.).
31	Reflect the difficulties of Chinese students in learning English language elements.
32	Integrate the content of Chinese culture and properly deal with the similarities and differences in cultural comparison.
33	Annotated in Chinese, concise, accurate and easy to understand.
34	The layout design (such as font size, mixed Chinese and foreign languages, layout, etc.) is clear, beautiful and functional.
35	Illustrations work closely with the content to help students understand the text.
36	The illustrations are clear and easy to understand, reflecting the sense of The Times and stimulating the creativity and imagination of learners.
37	According to learning needs, it is equipped with necessary supporting materials, such as workbooks, tests, word cards, wall charts, etc.
38	According to learning needs, it is equipped with digital learning resources, such as videos, supporting websites, apps, etc.
39	It is equipped with stage review materials and total review materials.

Table A2. Evaluation form for FLE, EFF and ENG

(adapted from Li et al., 2018; Jang et al., 2016; Lin et al., 2023)

**Foreign language enjoyment** (FLE)	Strong agreement	Moderate agreement	Uncertain	Moderate disagreement	Strong disagreement
I don't get bored during a foreign language class.					
I enjoy learning foreign languages.					
I've learnt interesting things in the process of learning foreign languages.					
In the class, I am proud of my foreign language grades.					
Learning foreign languages is fun.					
**Learning effectiveness** (EFF)	Strong agreement	Moderate agreement	Uncertain	Moderate disagreement	Strong disagreement
I feel my foreign language learning is efficient.					
I feel my foreign language learning is effective.					
I actively learn foreign language.					
I have many resources to learn foreign language.					
I think my foreign language learning leads to satisfactory performance.					
**Learning engagement** (ENG)	Strong agreement	Moderate agreement	Uncertain	Moderate disagreement	Strong disagreement
When I'm in a foreign language class, I listen very carefully.					
I feel interested in a foreign language class.					
I enjoy learning new things in a foreign language class.					
I get involved in a foreign language class.					
During a foreign language class, I express my preferences and opinions.					

During a foreign language class, I ask questions to help me learn.					
When learning about a new topic in a foreign language class, I usually try to summarize it in my own words.					
When reading in a foreign language class, I try to connect the ideas I am reading about with what I already know.					
